# Clinical heterogeneity and five phenotypes identified in pediatric Behçet’s syndrome: a cohort study from Shanghai Behçet’s syndrome database

**DOI:** 10.1007/s12519-023-00785-9

**Published:** 2024-02-05

**Authors:** Dan Hu, Chun-Hui She, Hua-Fang Bao, Jun Zou, Jian-Fei Cai, Jing‑Fen Ye, Yan Shen, Hai‑Fen Ma, Dan Luo, Jian-Long Guan

**Affiliations:** Department of Rheumatology and Immunology, Huadong Hospital Affiliated to Fudan University, 221 Yan’an West Road, 200040 Shanghai, China

**Keywords:** Behçet’s syndrome, Clinical manifestation, Cluster analysis, Pediatric, Phenotype

## Abstract

**Objectives:**

Behçet’s syndrome (BS) is a rare disease of unknown etiology, with limited reports especially in pediatric BS. The clinical characteristics and phenotypes of pediatric BS as a highly heterogeneous variable vessel vasculitis were investigated in this study.

**Methods:**

A cross-sectional study was conducted to compare clinical variables and descriptive characteristics of BS by age of onset and gender. Cluster analysis was then performed to identify the phenotypes of pediatric BS.

**Results:**

A total of 2082 BS patients were included in this study, 1834 adults and 248 children. Compared with adult-onset BS, pediatric BS had a higher incidence of folliculitis [relative risks (RR) and 95% confidence interval (CI) 1.3 (1.0–1.5)], uveitis of the left eye [RR and 95% CI 2.3 (1.0–5.0)], intestinal ulcer complications [RR and 95% CI 2.1 (1.1–4.2)], pericarditis [RR and 95% CI 2.5 (1.0–6.2)], and psychiatric disorders [RR and 95% CI 2.8(1.0–7.9)], while the incidence of thrombocytopenia was lower [RR 0.2 (0.1–1.0)]. Among pediatric BS, females had more genital ulcers, while males were more likely to have skin lesions, panuveitis, vascular involvement, venous lesions, cardiac involvement, and aortic aneurysms. Cluster analysis classified pediatric BS into five clusters (C1–C5): C1 (*n* = 61, 24.6%) showed gastrointestinal (GI) involvement; C2 (*n* = 44, 17.7%) was the central nervous system (CNS) type where 23 cases overlapped joint involvement; in C3 (*n* = 35, 14.1%), all patients presented with arthritis or arthralgia; all patients in C4 (*n* = 29, 11.7%) manifested ocular involvement, with a few patients overlapping with GI involvement or joint damage; C5 (*n* = 79, 31.9%) was the mucocutaneous type, presenting both oral ulcers, genital ulcers, and skin lesions.

**Conclusions:**

The clinical features of pediatric and adult BS differ significantly. Male and female pediatric BS also have a distinct demography. Five phenotypes including GI, CNS, joint, ocular, and mucocutaneous types were identified for pediatric BS.

**Graphical Abstract:**

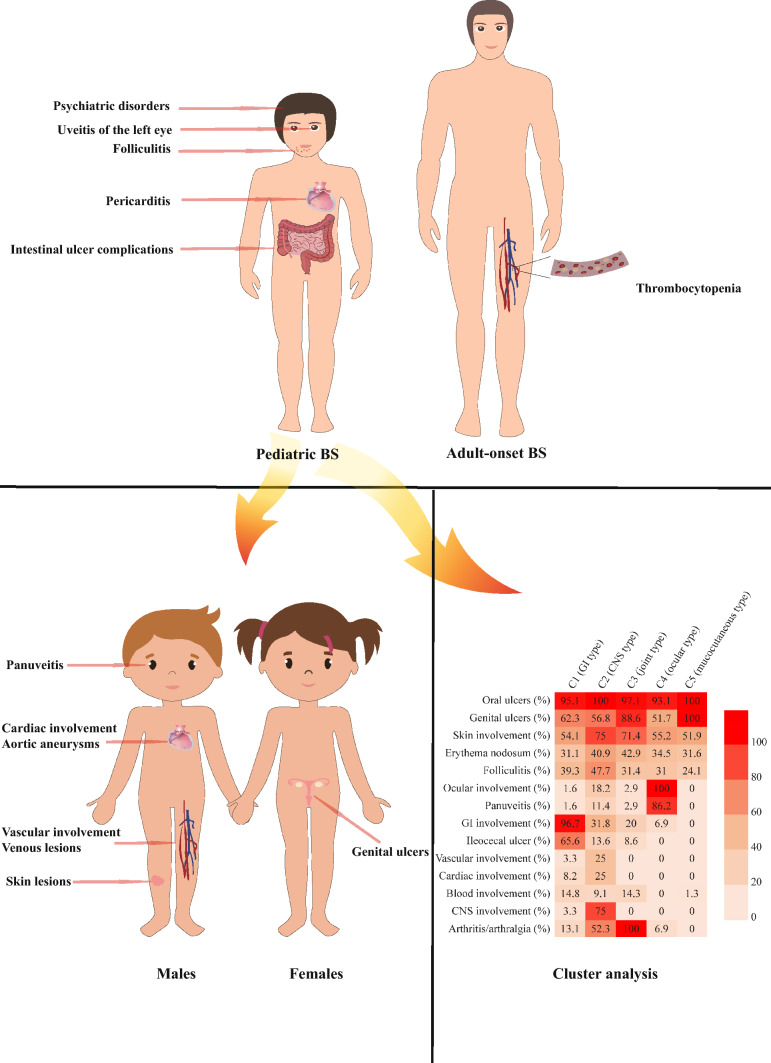

## Introduction

Behçet’s syndrome (BS) is a chronic systemic vasculitis characterized by mucocutaneous and ocular tissue involvement, as well as potential major organ damage affecting the vascular, gastrointestinal (GI) tract, articular, and neurological systems [1]. Major organ involvement in BS has significant heterogeneity and a unique geographical distribution [2]. The symptoms and major organ involvement often vary based on gender, age, and ethnicity [3, 4]. The highest incidence of BS occurs between the ages of 20 and 40 years, with a minority of cases (4%–26%) presenting before the age of 16 [5]. Therefore, BS is generally considered an adult-onset disease. While some patients may experience primary complaints in early childhood, only a small proportion meet diagnostic criteria by age 16 [6–8]. The true prevalence of pediatric BS remains unknown.

China is one of the endemic areas of BS [9], however, there is still a lack of epidemiological studies on BS, especially in pediatric BS [10]. Given the rarity and heterogeneity of this condition among children, epidemiological studies have produced inconsistent findings. Even within a single geographic area, results have varied across cohorts when comparing adults and pediatric patients [11, 12]. The diagnosis of BS relies on clinical manifestations, and the disease course may exhibit changes in clinical characteristics and severity. Therefore, epidemiological studies of BS hold great significance. Recently, Zou et al. proposed five adult phenotypes of BS: skin and mucosal, joint, GI, ocular, and cardiovascular involvement [13]. Additionally, three distinct phenotypes of pediatric BS were identified: mucocutaneous involvement, GI involvement, and a combination of uveitis with vascular and neurological symptoms [14]. Regarding the treatment of BS, the European League Against Rheumatism (EULAR) 2018 criteria noted that the treatment of BS should be individualized according to the patient's age, gender, and phenotype [15]. An accurate definition of phenotype clusters is crucial for disease treatment and management, as well as pathogenesis research. This study increases the number of cases and follow-up time in order to describe the characteristics of pediatric BS more accurately and guide the clinical diagnosis and treatment of pediatric BS.

## Methods

### Study subjects

A cross-sectional study was conducted on patients with BS who were admitted to the Department of Rheumatology and Immunology at Huadong Hospital Affiliated to Fudan University from September 2012 to March 2023. The International Criteria for Behçet’s Disease (ICBD) [16], Pediatric Behçet’s Disease (PEDBD) [17] and International Behçet’s Study Group (ISG) [18] were employed to assess the diagnosis of BS. Exclusion criteria comprised malignancy, infectious diseases, or other autoimmune disorders. In pediatric BS group, periodic fever, aphthous stomatitis, pharyngitis and cervical adenitis (PFAPA) syndrome should be considered and excluded [19]. Comprehensive records of clinical and laboratory data were scrutinized, encompassing demographic information, laboratory evaluations, imaging studies, and pathological findings. The definition of pediatric BS was an age of onset ≤ 16 years old, while adult BS referred to a diagnosis made after 16 years old. This study received approval from the Ethics Committee of Huadong Hospital, and all patients provided informed consent for participation.

### Clinical features and major organ involvement of Behçet’s syndrome

Organ involvement was evaluated through a comprehensive analysis of the patient's symptoms, medical record, physical examination, laboratory tests, imaging studies and endoscopic findings. Skin lesions encompassed erythema nodosum, folliculitis, pathergy test positive, erythema multiforme or thrombotic erythema [20]. GI manifestations of BS were confirmed through endoscopic findings, while intestinal tuberculosis and Crohn’s disease were ruled out [21]. Ocular involvement encompassed various forms of uveitis and ocular complications [22]. Vascular involvement was defined as deep vein thrombosis, large vein thrombosis, arterial thrombosis or aneurysm [23]. Cardiac lesions were confirmed through echocardiography or coronary angiography and computed tomography (CT) scans as endocarditis with valvular regurgitation, intracardiac thrombosis, ascending aortic aneurysm and coronary artery lesions [24]. CNS involvement includes parenchymal involvement and non-parenchymal manifestations of secondary vascular lesions [25]. Patients with hematological abnormalities had typical BS findings with thrombocytopenia or leukopenia, bone marrow abnormalities, or trisomy 8 [26]. Joint involvement includes arthralgia and arthritis. Arthritis was manifested as swelling of the joints of the extremities or joint ultrasound, CT or magnetic resonance imaging showed: arthritis, synovitis, joint destruction. Arthralgia was expressed as pain in the joints of the extremities or low back pain, but without joint swelling, inflammation, or destruction [27].

### Statistical analysis

SPSS 26.0 (IBM Corp, Armonk, NY, USA) software was used for statistical analysis. Continuous variables were presented as medians with 25%–75% interquartile ranges, while numerical variables were compared between groups using either Student’s *t*-test or Mann–Whitney *U* test. The chi-square test or Fisher’s exact test were employed for comparing categorical variables, with statistical significance set at *P* < 0.05. The two-step cluster analysis commenced by selecting variables that were classified as either continuous or categorical. The continuous variables comprised age at onset and disease course, while the categorical variables included gender, clinical characteristics (recurrent oral ulcers, genital ulcers, skin lesions, joint involvement), and major organ involvement (ocular, gastrointestinal, cardiac, vascular, hematologic and neurologic). A total of 13 variables were considered for cluster analysis. The log-likelihood method was employed to determine intersubjective distances and specific classifications.

## Results

### Comparison of clinical features between pediatric and adult-onset BS patients

We included a total of 2456 patients with BS, excluding 374 who did not meet the diagnostic criteria including incomplete BS, other autoimmune diseases such as systemic lupus erythematosus and Sjogren’s syndrome, and other gastrointestinal diseases such as Crohn’s and intestinal tuberculosis. The remaining 2082 patients met the diagnostic criteria. Of these, 1834 were adult patients and 248 were pediatric patients.

The median age of onset for pediatric patients was 12 years (interquartile range, IQR 8–14 years), with a median disease course of 15 years (IQR 9–21 years). The male to female gender ratio was 0.81:1.

Oral ulcer was the most common clinical manifestation, with a prevalence of 96.4%. Genital ulcers accounted for 79.3% of cases, while skin lesions were present in 56.7% of patients, including erythema nodosum (36.3%), folliculitis (27.9%), and pathergy test positive (5.4%). Arthritis or arthralgia was reported in 24.4% of cases. GI involvement was the most prevalent major organ involvement, accounting for 28.7% of cases, followed by ocular at 14.5%, and CNS involvement at 10.6%. Vascular involvement was observed in 6.9% of cases, with arterial involvement present in 58 cases and venous involvement present in 97 cases. Cardiac disease accounted for 8.1%. Hematological diseases accounted for 6.2%, with myelodysplastic syndrome (MDS) being present in 17 cases. Similar to adult BS, mucocutaneous involvement was the most prevalent manifestation in pediatric BS, while GI involvement (33.1%) was the most common major organ involvement. Vascular and cardiac involvements were rare occurrences in pediatric BS, accounting for only 5.2% and 6.5%, respectively. Detailed clinical characteristics are provided in Table 1.

**Table 1:** Comparison of clinical characteristics between pediatric and adult-onset BS patients

Variables	Total (*n* = 2082)	Pediatric patients (*n* = 248)	Adult patients (*n* = 1834)	Z/X^2^	*P*	RR (pediatrics:adults)
Age at onset (y)	30 (21–39)	12 (8–14)	31 (24–40)	25.594	**< 0.001**	–
Disease course (y)	10 (5–16)	15 (9–21)	10 (5–15)	8.775	**< 0.001**	–
Female	1073 (51.5%)	137 (55.2%)	936 (51.0%)	1.547	0.214	1.1 (1.0–1.2)
Oral ulcers	2008 (96.4%)	242 (97.6%)	1766 (96.3%)	1.058	0.304	1.0 (1.0–1.0)
≥ 1 time/mon	2685 (80.9%)	198 (79.8%)	1487 (81.1%)	0.218	0.641	1.0 (0.9–1.1)
≥ 1 times/season	323 (15.5%)	44 (17.7%)	279 (15.2%)	1.066	0.302	1.2 (0.9–1.6)
< 1 times/mon	64 (3.1%)	5 (2.0%)	59 (3.2%)	1.057	0.304	0.6 (0.3–1.5)
No oral ulcer	10 (0.5%)	1 (0.4%)	9 (0.5%)	0.035	0.825	0.8 (0.1–6.5)
Genital ulcers	1650 (79.3%)	188 (75.8%)	1462 (79.7%)	2.031	0.154	1.0 (0.9–1.0)
Skin lesion	1181 (56.7%)	148 (59.7%)	1033 (56.3%)	1.000	0.317	1.1 (0.9–1.2)
Erythema nodosum	755 (36.3%)	87 (35.1%)	668 (36.4%)	0.170	0.680	1.0 (0.8–1.2)
Folliculitis	580 (27.9%)	84 (33.9%)	496 (27%)	5.065	**0.024**	1.3 (1.0–1.5)
Pathergy test positive	113 (5.4%)	20 (8.1%)	93 (5.1%)	3.814	0.051	1.6 (1.0–2.5)
Erythema multiform	47 (2.3%)	6 (2.4%)	41 (2.2%)	0.033	0.855	1.1 (0.5–2.5)
Thrombophlebitis	12 (0.6%)	3 (1.2%)	9 (0.5%)	1.970	0.160	2.5 (0.7–9.0)
Ocular involvement	302 (14.5%)	39 (15.7%)	263 (14.3%)	0.338	0.561	1.1 (0.8–1.5)
Anterior uveitis	32 (1.5%)	2 (0.8%)	30 (1.6%)	0.993	0.319	0.5 (0.1–2.1)
Panuveitis	262 (12.6%)	32 (12.9%)	230 (12.5%)	0.026	0.872	1.0 (0.7–1.5)
Posterior uveitis	16 (0.8%)	4 (1.6%)	12 (0.7%)	2.632	0.105	2.5 (0.8–7.6)
Uveitis of the left eye	34 (1.6%)	8 (3.2%)	26 (1.4%)	4.446	**0.035**	2.3 (1.0–5.0)
Uveitis of the right eye	36 (1.7%)	3 (1.2%)	33 (1.8%)	0.447	0.504	0.7 (0.2–2.2)
Bilateral uveitis	163 (7.8%)	14 (5.6%)	149 (8.1%)	1.861	0.173	0.7 (0.4–1.2)
Retinal vasculitis	27 (1.3%)	6 (2.4%)	21 (1.1%)	2.771	0.096	2.1 (0.9–5.2)
GI involvement	597 (28.7%)	82 (33.1%)	515 (28.1%)	2.653	0.103	1.2 (1.0–1.4)
Upper digestive tract ulcer	199 (9.6%)	20 (8.1%)	179 (9.8%)	0.727	0.394	0.8 (0.5–1.3)
Small intestinal ulcer	42 (2%)	9 (3.6%)	33 (1.8%)	3.700	0.054	2.0 (1.0–4.2)
Ileocecal ulcer	328 (15.8%)	49 (19.8%)	279 (15.2%)	3.401	0.065	1.3 (1.0–1.7)
Ulcerative colitis	139 (6.7%)	21 (8.5%)	118 (6.4%)	1.450	0.228	1.3 (0.8–2.1)
Intestinal ulcer complications	45 (2.2%)	10 (4.0%)	35 (1.9%)	4.660	**0.031**	2.1 (1.1–4.2)
Vascular involvement	144 (6.9%)	13 (5.2%)	131 (7.1%)	1.226	0.268	0.7 (0.4–1.3)
Arterial	58 (2.8%)	5 (2.0%)	53 (2.9%)	0.616	0.433	0.7 (0.3–1.7)
Venous	97 (4.7%)	9 (3.6%)	88 (4.8%)	0.672	0.412	0.78 (0.4–1.5)
Cardiac involvement	169 (8.1%)	16 (6.5%)	153 (8.3%)	1.047	0.306	0.8 (0.5–1.3)
Aortic aneurysm	79 (3.8%)	4 (1.6%)	75 (4.1%)	3.670	0.055	0.4 (0.1–1.1)
Aortic regurgitation	99 (4.8%)	7 (2.8%)	92 (5.0%)	2.321	0.128	0.6 (0.3–1.2)
Mitral regurgitation	34 (1.6%)	2 (0.8%)	32 (1.7%)	1.197	0.274	0.5 (0.1–1.9)
Tricuspid regurgitation	14 (0.7%)	1 (0.4%)	13 (0.7%)	0.305	0.580	0.6( 0.1–4.3)
Myocardial infarction	18 (0.9%)	0 (0)	18 (1.0%)	2.455	0.117	-
Pericarditis	24 (1.2%)	6 (2.4%)	18 (1.0%)	3.964	**0.046**	2. 5 (1.0–6.2)
Blood involvement	129 (6.2%)	19 (7.7%)	110 (6.0%)	1.040	0.308	1.3 (0.8–2.0)
Leukocytopenia	82 (3.9%)	10 (4.0%)	72 (3.9%)	0.007	0.936	1.0 (0.5–2.0)
Thrombocytopenia	63 (3.0%)	2 (0.8%)	61 (3.3%)	4.726	**0.030**	0.2 (0.1–1.0)
MDS	17 (0.8%)	1 (0.4%)	16 (0.9%)	0.594	0.441	0.5 (0.1–3.5)
Trisomy 8	39 (1.9%)	1 (0.4%)	38 (2.1%)	3.310	0.069	0.2 (0.0–1.4)
CNS involvement	220 (10.6%)	35 (14.1%)	185 (10.1%)	3.746	0.053	1.4 (1.0–2.0)
Cerebral aneurysm	52 (2.5%)	5 (2.0%)	47 (2.6%)	0.268	0.605	0.8 (0.3–2.0)
Encephalopathy	34 (1.6%)	5 (2.0%)	29 (1.6%)	0.257	0.612	1.3 (0.5–3.3)
Mental disturbance	18 (0.9%)	5 (2.0%)	13 (0.7%)	4.356	**0.037**	2.8 (1.0–7.9)
Joint involvement	509 (24.4%)	68 (27.4%)	441 (24.0%)	1.346	0.250	1.1 (0.9–1.4)
Arthritis	265 (12.7%)	40 (16.1%)	225 (12.3%)	2.931	0.087	1.3 (1.0–1.8)
Arthralgia	244 (11.7%)	28 (11.3%)	216 (11.8%)	0.050	0.823	1.0 (0.7–1.4)

Bold characters indicate statistically significance

*BS* Behçet’s syndrome, *CNS* central nervous system, *GI* gastrointestinal, *MDS* myelodysplastic syndrome, – not available

We observed a higher incidence of folliculitis [relative risks (RR) and 95% confidence interval (CI) 1.3 (1.0–1.5)], uveitis of the left eye [RR and 95% CI 2.3 (1.0–5.0)], intestinal ulcer complications [RR and 95% CI 2.1 (1.1–4.2)], pericarditis [RR and 95% CI 2.5 (1.0–6.2)], and psychiatric disorders [RR and 95% CI 2.8(1.0–7.9)] in pediatric patients with BS compared to adult patients with the same condition; however, the incidence of thrombocytopenia decreased significantly [RR and 95% CI 0.2(0.1–1.0)]. Joint involvement and vascular involvement were not significantly different between the two groups.

### Gender-related clinical manifestations in pediatric Behçet’s syndrome

In terms of gender-related clinical features of pediatric BS, the prevalence of oral ulcers was highest in both sexes with no significant difference; however, females had a higher incidence of oral ulcers. Additionally, females exhibited an association with genital ulcers (*P* = 0.001) while males displayed associations with skin lesions (*P* = 0.043), panuveitis (*P* = 0.011), vascular involvement (*P* = 0.017), venous lesions (*P* = 0.042), cardiac involvement (*P* = 0.046), and aortic aneurysms (*P* = 0.025). No discernible differences based on sex were observed in other major organs involved (Table 2).

**Table 2:** Comparison of clinical manifestations between male and female pediatric BS patients

Variables	Total (*n* = 248)	Male (*n* = 111)	Female (*n* = 137)	Z/X^2^	*P*	RR (male:female)
Age at onset (y)	12.1 (8.7–14.4)	12.0 (8.5–14.5)	12.2 (8.7–14.2)	0.641	0.522	–
Disease course (y)	15.0 (9.3–21.0)	15.0 (9.0–20.0)	17.0 (10.0–22.0)	0.879	0.380	–
Oral ulcers	242 (97.6%)	109 (98.2%)	133 (97.1%)	0.325	0.569	1.0 (1.0–1.1)
≥ 1 time/mon	198 (79.8%)	82 (73.9%)	116 (84.7%)	4.441	**0.035**	0.9 (0.8–1.0)
≥ 1 times/season	44 (17.7%)	27 (24.3%)	17 (12.4%)	5.965	**0.015**	2.0 (1.1–3.4)
< 1 times/mon	5 (2.0%)	2 (1.8%)	3 (2.2%)	0.047	0.829	0.8 (0.1–4.8)
Genital ulcers	188 (75.8%)	73 (65.8%)	115 (83.9%)	11.045	**0.001**	0.8 (0.7–0.9)
Skin involvement	148 (59.7%)	74 (66.7%)	74 (54%)	4.079	**0.043**	1.2 (1.0–1.5)
Erythema nodosum	87 (35.1%)	42 (37.8%)	45 (32.8%)	0.671	0.413	1.2 (0.8–1.6)
Folliculitis	84 (33.9%)	42 (37.8%)	42 (30.7%)	1.412	0.235	1.2 (0.9–1.7)
Pathergy test positive	20 (8.1%)	12 (10.8%)	8 (5.8%)	2.044	0.153	1.9 (0.8–4.4)
Ocular involvement	39 (15.7%)	23 (20.7%)	16 (11.7%)	3.783	0.052	1.8 (1.0–3.2)
Anterior uveitis	2 (0.8%)	1 (0.9%)	1 (0.7%)	0.022	0.881	1.2 (0.1–19.5)
Panuveitis	32 (12.9%)	21 (18.9%)	11 (8%)	6.47	**0.011**	2.4 (1.2–4.7)
GI involvement	82 (33.1%)	36 (32.4%)	46 (33.6%)	0.036	0.849	1.0 (0.7–1.4)
Upper digestive tract ulcer	20 (8.1%)	7 (6.3%)	13 (9.5%)	0.838	0.360	0.7 (0.3–1.6)
Small intestinal ulcer	9 (3.6%)	2 (1.8%)	7 (5.1%)	1.918	0.166	0.4 (0.1–1.7)
Ileocecal ulcer	49 (19.8%)	22 (19.8%)	27 (19.7%)	0	0.982	1.0 (0.6–1.7)
Ulcerative colitis	21 (8.5%)	11 (9.9%)	10 (7.3%)	0.539	0.463	1.4 (0.6–3.1)
Intestinal ulcer complications	10 (4.0%)	6 (5.4%)	4 (2.9%)	0.979	0.322	1.9 (0.5–6.4)
Vascular involvement	13 (5.2%)	10 (9%)	3 (2.2%)	5.741	**0.017**	4.1 (1.2–14.6)
Arterial	5 (2.0%)	3 (2.7%)	2 (1.5%)	0.479	0.489	1.9 (0.3–10.9)
Venous	9 (3.6%)	7 (6.3%)	2 (1.5%)	4.18	**0.042**	4.3 (0.9–20.4)
Cardiac involvement	16 (6.5%)	11 (9.9%)	5 (3.6%)	3.982	**0.046**	2.7 (1.0–7.6)
Aortic aneurysm	4 (1.6%)	4 (3.6%)	0 (0)	5.018	**0.025**	–
Aortic regurgitation	7 (2.8%)	5 (4.5%)	2 (1.5%)	2.072	0.150	3.086 (0.61–15.601)
Hydropericardium	6 (2.4%)	5 (4.5%)	1 (0.7%)	3.701	0.054	6.171 (0.732–52.054)
Blood involvement	19 (7.7%)	7 (6.3%)	12 (8.8%)	0.521	0.47	0.72 (0.293–1.767)
CNS involvement	35 (14.1%)	14 (12.6%)	21 (15.3%)	0.373	0.541	0.823 (0.439–1.542)
Cerebral aneurysm	5 (2.0%)	4 (3.6%)	1 (0.7%)	2.563	0.109	4.937 (0.560–43.541)
Encephalopathy	5 (2.0%)	3 (2.7%)	2 (1.5%)	0.479	0.489	1.851 (0.315–10.886)
Mental disturbance	5 (2.0%)	1 (0.9%)	4 (2.9%)	1.265	0.261	0.309 (0.035–2.721)
Joint involvement	68 (27.4%)	31 (27.9%)	37 (27.0%)	0.026	0.872	1.034 (0.689–1.552)
Arthritis	40 (16.1%)	20 (18.0%)	20 (14.6%)	0.530	0.467	1.234 (0.700–2.176)
Arthralgia	28 (11.3%)	11 (9.9%)	17 (12.4%)	0.328	0.536	0.799 (0.390–1.634)

Bold characters indicate statistically significance

*BS* Behçet’s syndrome, *CNS* central nervous system, *GI* gastrointestinal, – not available

### Cluster analysis

Through cluster analysis, we identified five distinct clusters of pediatric BS and summarized their clustering characteristics in Table 3.

**Table 3:** Clinical characteristics of different clusters of pediatric BS

Variables	C1 (*n* = 61)	C2 (*n* = 44)	C3 (*n* = 35)	C4 (*n* = 29)	C5 (*n* = 79)	Total (*n* = 248)
Age at onset (y)	12 (10–14)	11.5 (8–14)	12 (8–14)	13 (8–14)	12 (9–14)	12 (9–14)
Disease course (y)	13 (8–20)	12.5 (8–22)	16.5 (12–25)	15 (10.5–23)	18 (10–24)	15 (9–21)
Female sex	34 (55.7%)	20 (45.5%)	20 (57.1%)	10 (34.5%)	53 (67.1%)	137 (55.2%)
Oral ulcers	58 (95.1%)	44 (100%)	34 (97.1%)	27 (93.1%)	**79 (100%)**	242 (97.6%)
Genital ulcers	38 (62.3%)	25 (56.8%)	31 (88.6%)	15 (51.7%)	**79 (100%)**	188 (75.8%)
Skin involvement	33 (54.1%)	33 (75.0%)	25 (71.4%)	16 (55.2%)	41 (51.9%)	148 (59.7%)
Erythema nodosum	19 (31.1%)	18 (40.9%)	15 (42.9%)	10 (34.5%)	25 (31.6%)	87 (35.1%)
Folliculitis	24 (39.3%)	21 (47.7%)	11 (31.4%)	9 (31.0%)	19 (24.1%)	84 (33.9%)
Pathergy test positive	5 (8.2%)	6 (13.6%)	3 (8.6%)	1 (3.4%)	5 (6.3%)	20 (8.1%)
Ocular involvement	1 (1.6%)	8 (18.2%)	1 (2.9%)	**29 (100%)**	0 (0)	39 (15.7%)
Panuveitis	1 (1.6%)	5 (11.4%)	1 (2.9%)	25 (86.2%)	0 (0)	32 (12.9%)
Anterior uveitis	0 (0)	0 (0)	0 (0)	2 (6.9%)	0 (0)	2 (0.8%)
GI involvement	**59 (96.7%)**	14 (31.8%)	7 (20.0%)	2 (6.9%)	0 (0)	82 (33.1%)
Upper digestive tract ulcer	14 (23.0%)	2 (4.5%)	3 (8.6%)	1 (3.4%)	0 (0)	20 (8.1%)
Small intestinal ulcer	5 (8.2%)	2 (4.5%)	2 (5.7%)	0 (0)	0 (0)	9 (3.6%)
Ileocecal ulcer	40 (65.6%)	6 (13.6%)	3 (8.6%)	0 (0)	0 (0)	49 (19.8%)
Colorectal ulcers	15 (24.6%)	3 (6.8%)	2 (5.7%)	1 (3.4%)	0 (0)	21 (8.5%)
Intestinal ulcer complications	8 (13.1%)	1 (2.3%)	1 (2.9%)	0 (0)	0 (0)	10 (4%)
Vascular involvement	2 (3.3%)	11 (25.0%)	0 (0)	0 (0)	0 (0)	13 (5.2%)
Arterial	1 (1.6%)	4 (9.1%)	0 (0)	0 (0)	0 (0)	5 (2.0%)
Venous	1 (1.6%)	8 (18.2%)	0 (0)	0 (0)	0 (0)	9 (3.6%)
Cardiac involvement	5 (8.2%)	11 (25.0%)	0 (0)	0 (0)	0 (0)	16 (6.5%)
Aortic aneurysm	2 (3.3%)	2 (4.5%)	0 (0)	0 (0)	0 (0)	4 (1.6%)
Aortic regurgitation	2 (3.3%)	5 (11.4%)	0 (0)	0 (0)	0 (0)	7 (2.8%)
Blood involvement	9 (14.8%)	4 (9.1%)	5 (14.3%)	0 (0)	1 (1.3%)	19 (7.7%)
CNS involvement	2 (3.3%)	**33 (75%)**	0 (0)	0 (0)	0 (0)	35 (14.1%)
Arthritis/arthralgia	8 (13.1%)	23 (52.3%)	**35 (100%)**	2 (6.9%)	0 (0)	68 (27.4%)

Bold characters indicate statistically significance

*BS* Behçet’s syndrome, *CNS* central nervous system, *GI* gastrointestinal, – not available

In Cluster1 (C1), the GI involvement type, a total of 61 cases were analyzed, with a gender ratio of 27 males to 34 females. The median age of onset was 12 years (IQR 10–14 years) and the median disease course was 13 years (IQR 8–20 years). GI involvement was observed in 96.7% of patients, including ileocecal ulcers in 40 cases, upper digestive tract ulcers in 14 cases, colorectal ulcers in 15 cases, and intestinal ulcer complications in five cases. Oral ulcers (95.1%), genital ulcers (62.3%), and skin lesions (54.1%) constituted the primary cases.

There were 44 cases identified in C2, the CNS involvement type, with a male-to-female gender ratio of 24 to 20. The median age of onset was 11.5 years (IQR 8–14 years) and the median disease course was 12.5 years (IQR 8–22 years). Related arthritis/arthralgia occurred in 23 cases. While all patients presented with oral ulcers; skin lesions accounted for three-quarters of the cases, and genital ulcers were observed in more than half of the patients.

A total of 35 cases were identified in C3, the joint involvement type, with a male-to-female gender ratio of 15 to 20. The median age of onset was 12 years (IQR 8–14 years) and the median disease course was 16.5 years (IQR 12–25 years). All patients presented with arthritis (*n* = 22) or arthralgia (*n* = 13) symptoms. Most cases had oral ulcers (97.1%), genital ulcers (88.6%), and skin lesions (71.4%). The involvement of other major organs was rare. GI involvement was only found in seven cases; ocular involvement was found in one case, and no vascular or cardiac lesions were observed.

In C4, the ocular involvement type, a total of 29 cases were included, with a male-to-female gender ratio of 19:10. The median age of onset was 13 years (IQR 8–14 years) and the median disease course was 15 years (IQR 10.5–23 years). All patients presented with ocular manifestations, including uveitis (27 cases), retinal vasculitis (4 cases), and vitreous opacity (8 cases). Oral ulcers were observed in 93.1% of patients, genital ulcers in 51.7%, and skin lesions in 55.2%. There were a few cases of intestinal involvement (6.9%) and joint lesions (6.9%), but no vascular or cardiac lesions.

**Fig. 1:**
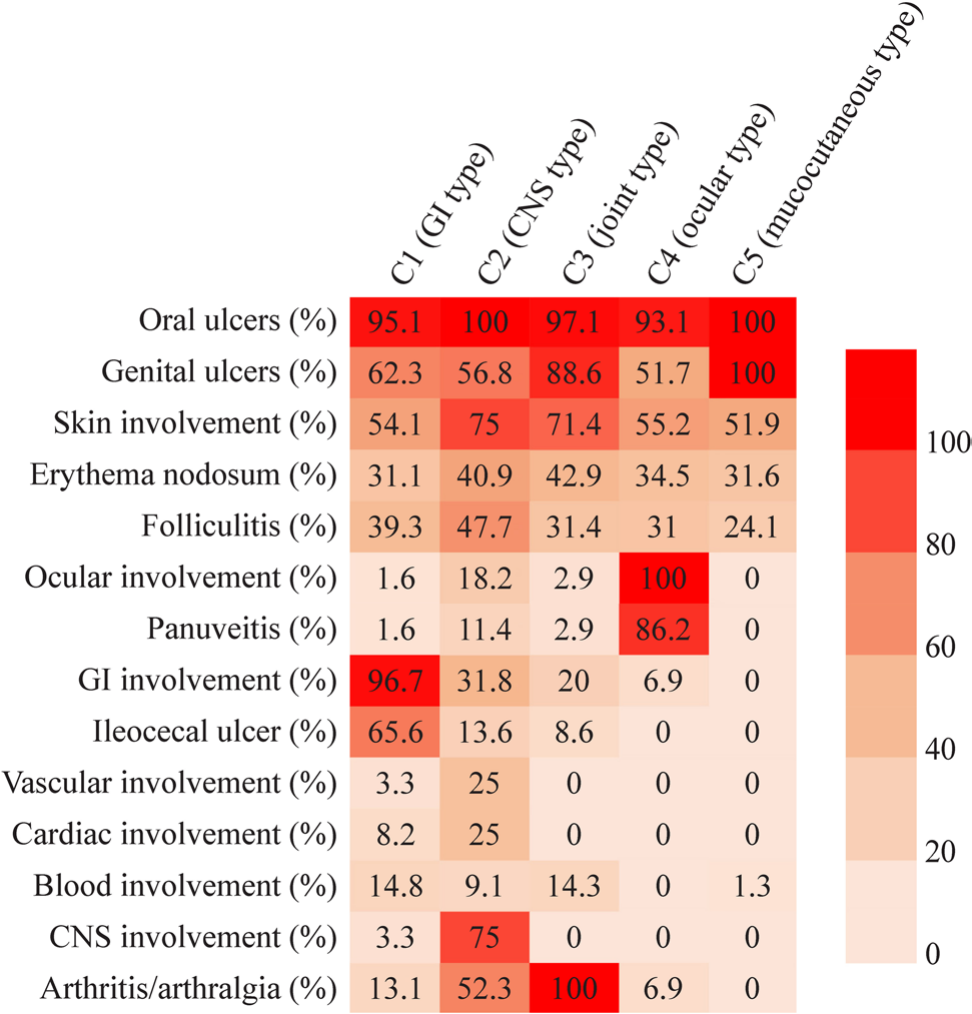
The features of five clusters of patients with pediatric BS. *BS* Behçet’s syndrome, *CNS* central nervous system, *GI* gastrointestinal

The largest proportion of cases were found in C5, the mucocutaneous involvement type, with 79 cases. The male-to-female gender ratio was 26:53 and the median age of onset was 12 years (IQR 9–14 years), while the median course of disease was 18 years (IQR 10–24 years). All patients presented with genital and oral ulcers, while skin lesions were observed in 51.9% of patients, erythema nodosum in 31.6%, and folliculitis in 24.1%. No other major organs were affected within this cohort. Figure 1 presents a summary of the clinical characteristics of these five categories of patients.

## Discussion

Our data suggest that mucocutaneous involvement is the most common manifestation of pediatric BS, while gastrointestinal involvement represents the most frequent major organ involvement, which is consistent with adult-onset disease [28]. However, Gezgin et al. [29] reported a higher prevalence of musculoskeletal symptoms (63%) than cutaneous manifestations (46%) in the Iranian cohort. Pediatric patients with BS had more folliculitis, uveitis of the left eye, intestinal ulcer complications, pericarditis, and psychiatric disorders, but less thrombocytopenia, compared with adult patients. Joint and vascular involvement did not show significant differences between the two groups, contradicting the study by Mastrolia et al. [8] in an Italian cohort. Their research concluded that there were no statistically significant discrepancies in mucocutaneous, ocular, CNS, and GI involvement between adult BS and pediatric BS patients. Additionally, joint manifestations were more prevalent in pediatric BS, while venous vascular events occurred more frequently in the adult cohort. Due to the increased sample size, our findings are consistent with previous studies [14] that indicate that female BS patients have a higher frequency of oral and genital ulcers. In addition to this, our study found that males were associated with skin lesions, panuveitis, vascular involvement, venous lesions, cardiac involvement, and aortic aneurysms. Therefore, male BS patients may exhibit more severe symptoms than their female counterparts in pediatric BS. Again, this is consistent with previous studies [30].

In this study, cluster analysis identified five phenotypic clusters of pediatric BS: GI, CNS, joint, ocular, and mucocutaneous involvement types. The largest proportion was accounted for by the mucocutaneous and GI types, which is consistent with previous studies [13]. However, unlike previous studies, the CNS, articular, and ocular types were classified into separate categories. Each subtype exhibited a relatively concentrated and predominant involvement of a single organ, with good clustering effects that are more consistent with clinical practice.

In this study, ocular involvement was found to be more prevalent in pediatric BS, which is consistent with the findings reported by Uziel et al. [31] However, Koné-Paut et al. [17] proposed that pediatric BS patients may have less ocular involvement than adult BS patients, but the involvement tends to be more severe. In studies of noninfectious uveitis, unilateral uveitis was more common, and the left eye was more affected (34.2%) than the right eye (25.4%) [32]. This study is the first to show that uveitis of the left eye is more common in pediatric BS than in adult BS. Studies have shown that male BS patients have a higher incidence and faster progression of uveitis [33, 34]. Panuveitis is also more common in males than females. We also confirmed the existence of this sex difference in pediatric BS. In cases of CNS-type pediatric BS, joint damage occurs in approximately half of all patients and there is a higher incidence of mental disorders compared to adult BS. Joint involvement often presents as an early symptom of the disease [35]. Studies have found that GI involvement is more common in pediatric BS [36]. Although our findings did not support this conclusion, our study suggested that the incidence of intestinal ulcer complications in pediatric BS patients was higher than that in adult BS patients. It cannot be excluded that missed or incorrect diagnosis of GI symptoms may have contributed to this trend. Given its multisystem nature, different clinical phenotypes of BS likely involve distinct mechanisms. Phenotypic clustering can facilitate disease stratification and ultimately enable precision treatment [37].

As is commonly known, the current diagnosis of BS relies on clinical symptoms and expert opinions due to the lack of specific laboratory indicators and histopathology. The diagnosis of pediatric BS poses even greater challenges given its rarity and insidious onset [38]. In addition, there are some differences in the treatment of BS patients in different regions [39]. In this study, we present a comprehensive description of the clinical features of BS in a larger cohort of Chinese juvenile patients. Building on previous studies that identified three distinct types of pediatric BS, our analysis reveals five phenotypic clusters that more accurately reflect the clinical reality. This precise phenotypic characterization provides an essential foundation for developing standardized treatment protocols for pediatric BS.

In conclusion, this study report the unique characteristics and five phenotypes for pediatric BS. These findings could be a useful step in the road toward personalized medicine for pediatric BS patients.

## Data Availability

Data are available upon reasonable request.
